# System Architecture and Operational Principles for a Configurable Clinical Decision Support System for Model-Informed Precision Dosing: Development, Verification, and Validation Study

**DOI:** 10.2196/95203

**Published:** 2026-11-12

**Authors:** Roberto Rigamonti, Yuan James Pétermann, Melvyn Herzig, Annie Ester Cathignol, Bruno Da Rocha Carvalho, Stellah George Mpagama, Bibie Said, Margaretha Laurent Sariko, Chantal Csajka, Monia Guidi, Yann Thoma

**Affiliations:** 1 School of Engineering and Management Vaud, HES-SO University of Applied Sciences and Arts Western Switzerland, Yverdon-les-Bains, Vaud, Switzerland, +41 24 557 62 73; 2 Centre for Research and Innovation in Clinical Pharmaceutical Sciences, University Hospital of Lausanne, Lausanne, Vaud, Switzerland; 3 Department of Clinical Pharmacology, Lausanne University Hospital and University of Lausanne, Lausanne, Vaud, Switzerland; 4 Faculty of Biology and Medicine, University of Lausanne, Lausanne, Vaud, Switzerland; 5 Kibong'oto Infectious Diseases Hospital, Sanya Juu, United Republic of Tanzania; 6 The Nelson Mandela African Institution of Science and Technology, Arusha, United Republic of Tanzania; 7 Kilimanjaro Clinical Research Institute, Moshi, United Republic of Tanzania; 8 Institute of Pharmaceutical Sciences of Western Switzerland, University of Geneva and University of Lausanne, Geneva & Lausanne, Switzerland; 9 School of Pharmaceutical Sciences, University of Geneva, Geneva, Switzerland

**Keywords:** therapeutic drug monitoring, model-informed precision dosing, imatinib, clinical decision support system, point-of-care testing

## Abstract

**Background:**

Therapeutic drug monitoring (TDM) supports individualized pharmacotherapy, yet its implementation in routine care remains limited by operational constraints, data quality issues, and the need for expert interpretation. Model-informed precision dosing (MIPD) addresses several of these limitations through population pharmacokinetic modeling and Bayesian forecasting, but its effective deployment in clinical practice still requires human TDM experts.

**Objective:**

This paper presents Tucuxi-CDSS, a configurable and open-source clinical decision support system (CDSS) designed to enable safe, standardized, and automated MIPD-guided dose individualization, with a particular focus on safeguarding against pre-analytical and data entry errors.

**Methods:**

We detail the Tucuxi-CDSS system architecture, its data validation workflow, decision logic, and configuration mechanisms. The system is built around extensive configurability through XML-based drug files, language files, and reporting templates. Verification was performed through unit testing, system-level tests, and random input fuzzing, while validation was conducted by comparing CDSS outputs against those of a human TDM expert in an *in silico* study.

**Results:**

The resulting system supports end-to-end MIPD workflows, from data import and automated error detection through dosage adaptation and clinical report generation. An implementation study for rifampicin dosing optimization in routine tuberculosis care is currently underway in Tanzania, and an illustrative use case with imatinib demonstrates the system’s covariate handling and reporting capabilities. We also briefly summarize the *in silico* validation against a human TDM expert across 60 virtual patients: The CDSS matched the expert in error detection, with numerically higher sensitivity (53% versus 45%) but lower specificity (83% versus 100%), and it achieved higher therapeutic target attainment (55% versus 37%; *P*=.046). These gains came at the cost of 4 detrimental interventions in the CDSS arm, against none for the expert.

**Conclusions:**

Tucuxi-CDSS provides a modular, extensible, and open-source platform intended to extend structured, reproducible TDM oversight to settings where trained pharmacologists are scarce. Its full performance evaluation against a TDM expert is presented in a companion paper and supports future prospective studies on CDSS adoption in clinical practice.

## Introduction

Therapeutic drug monitoring (TDM) is a cornerstone of individualized pharmacotherapy, providing an approach to tailor dosing best suited to the patient using individual measured drug concentrations typically in matrices such as blood or plasma [1-3]. By relating observed levels in blood or plasma to established therapeutic targets derived from pharmacokinetic/pharmacodynamic (PK/PD) relationships, TDM experts secure efficacy while limiting toxicity. In this sense, TDM operationalizes the precision medicine paradigm at the bedside, providing a dynamic, phenotypic view of drug exposure across several medical fields such as oncology, infectious diseases, immunology, or psychiatry [1-3].

Despite its benefits, traditional TDM is often constrained by significant operational limitations. Conventional approaches frequently rely on interpretation of sparse data, necessitating rigid adherence to steady-state sampling timings, most notably trough levels [1,2,4]. Dose adjustments in this context are often derived from a rudimentary “rule-of-three” law, which may oversimplify the PK reality of some drugs [2,4]. Indeed, although appropriate for compounds with linear PK, this proportional approach is fundamentally unsuited for drugs exhibiting nonlinear kinetics, where proportional dose adjustments can cause disproportionate shifts in exposure, either exceeding toxic thresholds or falling below therapeutic targets [2]. It is also not well-suited for other types of targets such as the area under the curve or, in antimicrobial pharmacology, the fraction of the dosing interval during which the free-drug concentration exceeds the minimum inhibitory concentration. Moreover, the existing workflow is often fragmented and cumbersome across multiple clinical stages, including sample collection, transportation, and laboratory analysis, making it highly susceptible to errors such as mistimed samples or data entry inaccuracies [3,5]. Timing inaccuracies constitute a major cause of errors: Up to 60% of drug concentrations are drawn at inappropriate sampling times, and 22% to 31% are collected prior to achieving steady state, further complicating interpretation by TDM experts [5]. These timing discrepancies combined with the frequent omission of critical data, such as precise dosing histories or current regimens, significantly undermine the reliability of resulting therapeutic recommendations, thereby hindering the timely delivery of precision dosing in routine care [5]. In addition, a significant lack of knowledge about TDM, including low awareness of its existence and basic principles, further impedes proper adaptation of the approach [6].

The emergence of model-informed precision dosing (MIPD) addresses these limitations by integrating population pharmacokinetic (popPK) models and Bayesian forecasting into the TDM process [4,7]. PopPK models characterize the typical PK behavior of a drug within a studied population while quantifying inter- and intra-individual variability. These models often incorporate patient-specific covariates (such as age, body weight, or renal function) to explain systematic differences in drug PK across individuals [4]. Although these models enable *a priori* dosing based on available individual covariates, the framework is most effective when using the more accurate Bayesian *a posteriori* forecasting, which integrates individual measured drug concentrations [8]. These approaches allow for the prediction of an individual’s exposure markers without the strict requirements of steady-state conditions or strict sampling timing [8]. MIPD accommodates nonlinear or time-varying kinetics, enhancing the flexibility and accuracy of dosing adjustments in complex clinical environments where traditional sampling is often impractical [4]. Despite its theoretical advantages, the integration of MIPD into routine clinical practice is hindered by significant operational barriers. A fundamental challenge is the inherent vulnerability of these models to erroneous or missing data; without expert oversight to scrutinize inputs, inaccuracies such as erroneous time stamps, implausible concentration measurements, or incomplete dosing records can prevent the mathematical framework from operating in the best of cases or lead to dramatic recommendations in the worst cases. This “garbage in, garbage out” risk can result in inappropriate or even hazardous therapeutic recommendations [5]. Furthermore, the technical complexity of standalone MIPD platforms presents a substantial barrier to adoption for nonexpert clinicians. This challenge is particularly acute in decentralized or resource-constrained settings where TDM specialists are unavailable and clinicians must operate independently [6,9]. In these contexts, the absence of expert-led quality control necessitates a transition from systems that merely perform computations to those that actively validate data. This requirement underscores the need for an integrated software layer capable of detecting errors and ensuring the clinical safety of model-derived advice.

To fully operationalize the potential of MIPD within routine care, clinical decision support systems (CDSSs) provide a critical architectural layer of validation and decision logic. Although MIPD can be broadly categorized as a form of CDSS by supporting clinicians’ decision for dosing adjustments, the term CDSS is specifically reserved here for the integration of an MIPD engine with a robust, rule-based validation system [10-12]. In settings where TDM experts are lacking, such a form of CDSS stands as the closest substitute, scrutinizing input data, including sampling time stamps, the dosing history, and measured concentrations, to identify and flag inconsistencies before providing a clinical recommendation. By checking data plausibility and identifying potential reporting errors, the system ensures that the quantitative outputs of MIPD are translated into clear, standardized, and actionable clinical recommendations. A well-designed CDSS properly integrated in the clinical workflow can significantly reduce preventable medication errors and shorten the interval between sample measurement and therapeutic decision [10,12]. Furthermore, by managing intelligent alert thresholds, these systems can detect true clinical problems while minimizing “alert fatigue,” thereby maintaining clinician trust and ensuring the sustainable adoption of TDM at the bedside [11,12].

Fully embedded in clinical initiatives, the development of the proposed CDSS has been driven by 2 distinct projects that address different TDM challenges and health care environments. The CDSS originally stemmed from the TuberXpert project, which focuses on optimizing rifampicin dosing for tuberculosis (TB) treatment in resource-limited settings, specifically Tanzania [13]. Rifampicin presents a significant challenge for traditional TDM due to its complex nonlinear kinetics, which renders standard proportional dose adjustments inaccurate [2]. In the Tanzanian setting, where there is a critical absence of pharmacologists and established TDM infrastructure, the CDSS serves as a foundational tool that enables the implementation of TDM by providing structured, reproducible TDM oversight and data validation [13]. In contrast, the OncoPok project applies the system within an oncology context where imatinib, a drug with simpler linear kinetics, is used [13-16]. Although specialized experts are available in this setting, the project focuses on decentralized, bedside monitoring enabled by point-of-care (POC) biosensor technology [17]. This real-time application requires a streamlined interface that automates data control and delivers rapid, actionable recommendations directly to clinicians. For this application, the CDSS is configured to assist with maintaining therapeutic trough concentrations within the target range of 1100 µg/L to 2000 µg/L [16,18]. Throughout this paper, drug concentrations are expressed in µg/L. The figures and the generated report render this unit in the application’s plain-ASCII form, ug/L. Two popPK models are available for imatinib. The first targets a trough range of 1100 µg/L to 2000 µg/L and is the one applied in [19], in order to comply with the deploying institution’s guidelines. The second, the model of Gotta et al [20], targets a range of 750 µg/L to 1500 µg/L and is the one used in this paper, because it carries the patient covariates and is therefore more instructive for demonstrating the software. The range applied by Tucuxi-CDSS in a given deployment is declared in the drug-model configuration file. This coexistence also illustrates how readily the system is customized to the internal guidelines of each institution.

This paper describes the architecture, operational workflow, verification strategy, and configurability of Tucuxi-CDSS and reports the outcomes of its verification together with a summary of its validation against a human TDM expert, the full study of which is presented in the companion paper [19]. It especially targets non-TDM experts, while the main *in silico* study discussing its safety and performance for imatinib administration in comparison to a human TDM expert is presented elsewhere [19]. Although originally conceived for the more complex pharmacological scenario of the TuberXpert project, this work uses imatinib as a more straightforward case study to highlight the system’s core functionalities and architectural robustness.

In particular, this paper focuses on the computational steps underlying dose individualization and the generation of clinical recommendations, mechanisms for detecting data errors, and configurations used to define drug-specific behavior and safeguards, with a description of the ongoing rifampicin implementation study within TuberXpert and an illustrative use case focused on the adjustment of imatinib dosages.

## Methods

### System Description

Tucuxi-CDSS is an open-source, cross-platform CDSS system that has been designed from its inception with a strong emphasis on extensibility, including the support of multiple languages and institutional practices. Figure 1 gives an overview of the computational steps performed by Tucuxi-CDSS.

Given patient-specific information (dosage history, samples, and covariates) and a drug model, after an error detection step, the system computes a suggested dosage adaptation using the Tucuxi-Core software, derives an adjustment recommendation, and draws a report for the clinician. The drug models used by Tucuxi-CDSS are directly derived from Tucuxi and detail the drug popPK model, possible doses and dosing intervals, and desired therapeutic targets [21]. The drug files themselves are maintained in a dedicated open-source repository [22] distinct from that of the system code, so that models may be reviewed, reused, and extended independently of the software that consumes them. Each model specifies how patient-specific data (covariates) are integrated in the evaluation and expresses the inter- and intrapatient variability.

The following sections delve into the details of the architecture, the operational workflow, and the configurability of the system.

**Figure 1.**
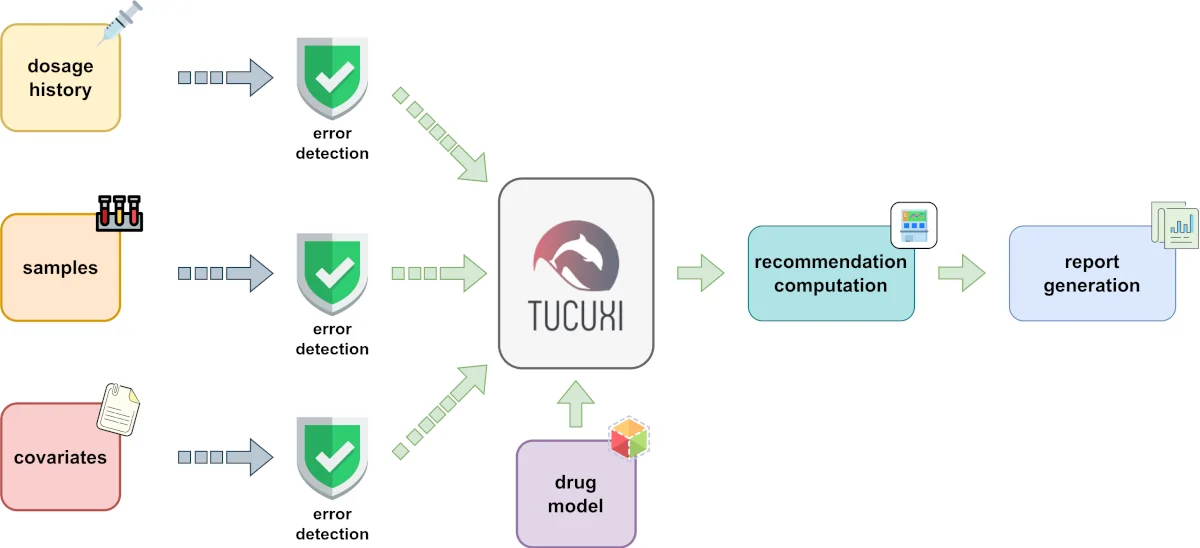
Overview of the computational steps performed by Tucuxi-CDSS. The patient’s dosage history, associated concentration samples, and covariates are first screened for pre-analytical and data entry errors then passed, together with the selected drug model, to the Tucuxi-Core computing engine. The resulting dosage recommendation is formatted into a report for the practitioner.

### Architecture

Tucuxi-CDSS is a modular system that acts as a control and interface layer to the Tucuxi-core computing engine, validating and preprocessing the input data, requesting the desired computations, and finally interpreting the computation results and formatting them according to user preferences [23]. It contains 3 processing layers: (1) data validation and preprocessing, (2) model-based computation, and (3) decision logic and reporting.

In the data validation and preprocessing layer, administered doses and sampled concentrations undergo a first validation step in which trivial data entry errors (eg, sample dates that precede the treatment start) are detected. If no clear error is identified, drug-specific checks are performed next (eg, the sample times after the dose are checked against the drug’s half-life, and a warning is raised if they are not representative enough for the model). This condition might stem from either a poorly applied medical protocol (eg, sample timing not duly respected) or, more likely, once more from data entry mistakes. Those pre-analytical errors have a high impact on the TDM process, and an automated system for their early identification is expected to yield significant benefits for both patients’ safety and quality assurance [5].

The model-based computation layer uses the selected drug model. Input data are structured in a format suitable for processing with the Tucuxi-Core computational engine to perform the required computations. The system performs a statistical analysis on the received samples data and derives their likelihood, taking into account the percentiles of predicted concentrations for the typical patient. Computation of these percentiles is performed by Tucuxi-Core based on the popPK model of the selected drug. This allows further validation of the input data, as it flags concentrations that are statistically unlikely and might result from data entry errors. If requested, Tucuxi-Core can also expose goodness-of-fit statistics for the computed data, to allow the practitioner in charge of delivering the treatment to further assess the quality of the results.

The decision logic and reporting layer is the third layer. Among Tucuxi-CDSS objectives is its use as TDM support in regions where no TDM experts are readily available, so a clear interpretation of the computed concentrations and clinical recommendation are mandatory components of the CDSS. Clinical recommendations are generated by comparing predicted exposure metrics to target ranges defined in the Tucuxi configuration drug files [21]. These recommendations, besides considering drug-specific criteria, must also consider user-selectable constraints (eg, certain dosage regimens are atypical in some institutions or countries, whereas they are common practice in others).

Tucuxi-CDSS is implemented as a command-line tool and can therefore be easily integrated into a pre-existing workflow. For instance, it can be accessed from a web app, directly returning the results in HTML form, or can be invoked by the graphical user interface of Tucuxi. Furthermore, since it is implemented in the C++ language, it can be linked as an external library into existing software.

### Operational Workflow

#### Data Import and Error Detection

The workflow starts by importing all data required for the process, which contain, in addition to information about which drug and drug model must be considered, patient- and physician-related data. The processing steps are further customized by loading the different configuration files.

A consistency check is made to ensure that the formulation and routes are supported by the selected model, and the covariate values are checked to ensure that they are within the boundaries specified in the configuration drug file and suited for the selected model. Boundary violations can either be soft or hard, depending on whether a lower or a higher threshold is exceeded: The former simply raises a warning that will be shown in the final report, while the latter will result in an error message. An example could be the age of the patient: If a given drug model is valid for patients younger than 80 years, then a soft threshold could be set to 82 years to ensure that the processing is still performed but a warning is displayed. That way, the practitioner can be warned of the validity of the results. A hard threshold could be set to 110 years, since it is very unlikely that a patient of that age shows up, and it is more likely that the age is the result of a data entry mistake. If a covariate required by the drug file is not specified for a given patient, a default value specified in the drug file (usually the median of the population) is considered, but a warning message is emitted.

In the next step, administered doses are checked to be within a range specified in the drug file, and dose dates are explicitly expressed from shorthands that are used to make data entry more manageable. For instance, a dose taken at 8 AM every day between 2 dates can be expressed as such, instead of explicitly writing each individual dose. Samples are also checked to lie within 10 to 90 percentiles or 1 to 99 percentiles of the population, since violations could hint at either data entry errors or a severely compromised treatment. Typically, within the 10 to 90 percentiles the sample is considered normal. If within 1 to 10 percentiles or 90 to 99 percentiles, a warning is issued, and if outside the 1 to 99 percentile, then a stronger warning is issued, as it is likely to be an error. The CDSS can only operate on the given data and has no direct way of assessing whether the data are correct or not besides informing the practitioner of the limited plausibility (in statistical terms) of the observed values. In the Bayesian framework of Tucuxi-Core, the deviation between the observed sample value and the concentration predicted for the typical patient is the quantitative counterpart of the percentile-based warning; Tucuxi-CDSS surfaces it qualitatively through soft and hard percentile thresholds (Table 1).

**Table 1.** List of checks automatically performed by Tucuxi-CDSS to spot data entry or methodological errors and to validate all possible entries that the medical staff will input to locate implausible values.

Check	Error type	Description
Covariate values	Data entry/model	Covariate values must fit within the boundaries specified by the drug file.
Missing covariate	Model	Missing values are replaced by default values, but a warning is emitted.
Valid doses	Data entry/model	Doses must be valid (within the range of doses as available in the drug file).
Sample dates	Data entry/procedure	Sample dates are checked to be plausible (not before treatment start and no more than 5 half-lives after last dose) and to be meaningful according to the dosage adjustment (better not to be during the absorption phase).
Therapeutic targets	Data entry	Targets are checked to be noncontradictory.

A final check is performed to ensure that the sample and dose dates are compatible with the drug specifications and that the requested therapeutic targets are not contradictory. By default, these targets are extracted from the drug model, but the user can override these values if requested for a specific patient. Therefore, the system has to ensure that, if multiple targets are supplied, they do not lead to contradictions (eg, 2 targets as trough concentrations with different values).

The list of checks performed by the system is summarized in Table 1.

#### Adaptation Computation and Report Generation

The computational core is then queried to perform the therapeutic drug analysis. This process is based on concentration predictions exploiting maximum *a posteriori* PK parameter estimation. This method, at the core of Bayesian forecasting, allows estimating the most likely PK parameters based on the current dosage history, covariates, and observed concentrations. Knowing these parameters enables the computation of various potential dosages, which results in expected concentrations and the corresponding adjustment suggestion to meet the desired therapeutic targets by appropriately combining allowed values for unitary doses and dosing intervals [23]. In particular, the software starts by predicting the concentrations when no change to drug dosage is performed. The result is checked against the target, and the process is repeated for several allowed changes. Only appropriate dosage changes (that is, changes that do not leave the target region) are retained.

These changes are further investigated to check whether a transient modulation (which only affects the next dose of the new regimen) could improve the convergence to the target.

Among the candidates, the one with the highest score of adjustment quality is chosen. Remotely inspired by the *r*² statistic, this score is computed, for each target defined for the drug, as:


$$Score\;=\;1-\frac{{\left(\text{Log}\left(C_{predicted}\right)-\text{Log}\left(C_{target}\right)\right)}^{2}}{{\left(0.5\text{Log}\left(C_{up}\right)-0.5\text{Log}\left(C_{lo}\right)\right)}^{2}}$$

and is then averaged over the targets. Here, C_predicted is the value predicted at steady state for the quantity in which the target is expressed, being the trough concentration, peak, mean level, area under the curve, or cumulative area under the curve according to the drug model; C_target is the target level; and C_up and C_lo are the upper and lower limits of the therapeutic interval. When the reference literature reports only the interval, C_target is taken by default as the geometric mean of its limits. When C_predicted falls outside the [C_lo,C_up] range, the candidate is rejected. The denominator is the relative radius of the therapeutic interval on a logarithmic scale, so the score of the accepted ones equals one when the prediction hits the target exactly and decreases as it gets farther from it. Because both terms are logarithms, the base is immaterial. Working on a logarithmic scale renders the score symmetric under multiplicative deviations from the target, which is the natural scale for concentration exposures.

Data are finally collected and, once a justification has been assembled from a set of standard sentences, a report is created by filling the chosen institutional template. The direction stated in the justification is determined by the change in overall exposure, each regimen being normalized to a common daily basis so that the unit dose and the dosing interval are described as the mechanism of the recommendation rather than as its effect. Elapsed postdose time intervals displayed in the report are shown as hours and minutes (for example, 1h45m), rendered through a single formatter for consistency.

### Configurability

The heavy customizability of Tucuxi-CDSS is due to the extensive use of XML configuration files. Besides adapting the drug files themselves to account for institution-specific protocols, an additional configuration file sets boundaries on the different covariates (eg, weight or age) to ensure that only appropriate models are used for the predictions and to tailor it to the specific population. Furthermore, computational limits can be imposed on the different tasks to ensure that the load matches the available computing resources.

Language files are given to allow the translation of warnings, user messages, and clinical recommendations. Adding a new language simply requires translating the content of an XML file with the sentences that are then automatically retrieved by the software once the given language is selected. The same mechanism allows an institution to reword the existing sentences to suit local usage, without touching the code.

Finally, the reporting templates (in HTML format) can be easily customized to fit the institutional requirements.

### Verification and Validation

Tucuxi-CDSS has undergone a test campaign structured on 3 different levels: In addition to typical unit tests (developed in C++ with the GoogleTest framework and aimed at validating the behavior of the individual components), complete system tests have been manually performed on predefined use cases based on imatinib and for which the expected result was known in advance. A custom fuzzing tool was also developed in Python to test the robustness of the software, detecting crashes when random changes to the values or tags of the input XML file are performed. In particular, the fuzzer starts from a valid XML input describing a fictitious patient, and random errors are introduced (eg, ambiguous or invalid dates are set, measurement units are removed) to either trigger a software crash or have an unexpected behavior. The command line tool is then invoked, and the behavior of the system is logged to a file for subsequent investigation. The fuzzer was run repeatedly over the course of development. The defects it surfaced were errors in the parsing of malformed input, and each was corrected as it arose; no crash of the command-line tool survived the current test suite. The fuzzer, as well as the 2 other levels of tests, forms part of the open-source repository [24] and may be run against the current code base by any developer.

Although verification is meant to check that the system “does the thing right,” validation consists of assessing whether it “does the right thing.” Validation has therefore been carried out by comparing the CDSS outputs with those of a human TDM expert in an *in silico* study centered on imatinib, to rigorously evaluate the correctness and safety of the system. The virtual patients were generated *in silico* from a population PK model of imatinib that includes no covariates, so that the simulated concentrations depend upon the dosage history alone; the generation procedure is described in the companion paper [19]. The data were organized in a spreadsheet and gave for each patient a treatment start date, a dose and dosing interval, the date of the last intake, and a single sample date and value. Data quality errors (such as timing inaccuracies and reporting errors) were purposefully introduced to stress-test the resilience of the system and compare its effectiveness in spotting them compared with that of a human expert. The CDSS was fed the data through a Python script that parsed the spreadsheet and built the corresponding XML input data file. This parsing step inevitably performed an initial error detection. For instance, input data files for patients for which the last intake preceded the treatment start date could not be created. The script also invoked, for each patient, the CDSS executable; parsed the output XML report for warnings, errors, and the suggested adaptations; and finally entered the results in another spreadsheet.

Note that data generation was performed by a person outside the development team, so the developers were unaware of the presence of errors in the input data and their type. The full methodology and results are presented in the companion paper [19].

For completeness, we summarize the outcome of that validation here. Across 60 virtual patients enriched with pre-analytical errors, Tucuxi-CDSS matched the human TDM expert in error-detection capability, showing numerically higher sensitivity (53%, 95% CI 35%‐70%, vs 45%, 95% CI 30%‐61%) and lower specificity (83%, 95% CI 63%‐95%, vs 100%, 95% CI 81%‐100%), and it achieved a significantly higher rate of therapeutic target attainment (55% vs 37%; *P*=.046). Four detrimental interventions were observed in the CDSS arm, against none in the expert arm. In one further case, the patient drifted out of the therapeutic range at steady state in both arms, no adaptation having been possible. The full methodology, complete statistics, and safety analysis are reported in the companion paper [19].

### Ethical Considerations

This manuscript reports the design, operational principles, and verification strategy of a software system. It does not involve the collection nor the secondary analysis of human subject data. The imatinib case used in the Results section to illustrate the report is entirely fictitious and was constructed for demonstration purposes only; it corresponds to no identifiable patient. This work falls outside the scope of the Swiss Federal Act on Research involving Human Beings (Human Research Act), which governs research involving human beings, biological material of human origin, or health-related personal data; accordingly, no application to a cantonal research ethics committee was required. The popPK model from which the virtual patients of the validation study were simulated had been developed in a separate study from clinical data collected in accordance with institutional guidelines at Lausanne University Hospital and approved by the ethics committee of the canton of Vaud (project number 2024‐01723). Informed consent was obtained from all patients through the institution’s general consent framework. The rifampicin implementation study referenced in the Results section is conducted within the TuberXpert project, whose protocol, ethics approvals, and informed consent procedures are described in the TuberXpert protocol paper [13]; it received ethical approval from the local health research committee (reference KNCHREC003) and from the National Health Research Committee of Tanzania (reference NIMR/HQ/R.8a/Vol.IX/2988). All figures included in the manuscript are synthetic or fictitious and contain no personally identifiable information.

## Results

### Deployment Example: Routine Clinical Use of CDSS for Rifampicin Optimization in TB Care

Tucuxi-CDSS was originally developed to optimize rifampicin dosing in routine TB care in resource-limited settings. An early-phase implementation study is currently underway in Tanzania to evaluate the integration of the CDSS into real-world clinical workflows [13]. This phase focuses on collecting high-quality clinical, PK, and contextual data to inform and refine the dosing model, particularly for patients with common comorbidities such as HIV infection, diabetes, and undernutrition; those are conditions that frequently coexist in resource-limited settings and significantly influence rifampicin exposure [2]. The system architecture integrates patient-level data inputs, MIPD algorithms, and clinician-facing decision outputs to support individualized treatment recommendations. Successful implementation and validation of the CDSS in routine practice will generate critical evidence on feasibility, safety, and effectiveness, thereby supporting its broader integration into national TB care programs in TB endemic settings. Recruitment and data collection are ongoing; no clinical outcome is reported here.

### Imatinib Report Example

Because the drug model used in the *in silico* evaluation includes no covariates, as noted in the Methods section, we constructed a purely illustrative fictitious case using the imatinib model from Gotta et al [15,20] in order to exercise the covariate-handling capabilities of the system. This case is intended solely to walk the reader through the outputs produced by Tucuxi-CDSS, and it does not, in any sense, constitute a clinical validation of the system. Clinical validation, based on an *in silico* study against a human TDM expert, is reported in the companion paper [19].

Starting from a fictitious patient who had a daily imatinib intake of 450 mg for about 1 month and 2 samples taken, the resulting report is given in Figure 2, while the corresponding drug exposure and drug blood concentration diagrams are given in Figures 3 and 4, respectively. In the left column of the report, in addition to the administrative data, we can observe the patient covariates required by the model, including the GIST (gastrointestinal stromal tumor) covariate, and the related warnings. In particular, the clinician did not complete the sex field, and the weight of the patient exceeds the limits for the used drug model. The column on the right starts by summarizing the current dosage and monitoring history. Since the second sample was taken at an inappropriate time, a detailed warning message indicates that it might not be very representative, even though the observed value is still plausible for the given dosage. Below that, the trough concentration is compared with the target, and the patient is clearly underdosed. Indeed, the dosage recommendation increases the overall daily exposure, moving from 450 mg every 24 hours to 400 mg every 12 hours (from 450 to 800 mg per day), thereby raising the trough concentration while limiting the peak-to-trough excursion. The recommendation also includes a single loading dose of 600 mg, administered before the new regimen begins. This loading dose is the transient modulation described in the Methods section, applied to the first dose of the new regimen to accelerate convergence to the target trough. A justification explains why such a strategy has been adopted. Tucuxi-CDSS computes the recommendation while leaving the final decision to the clinician. The expected results of the adoption of the new dosage regimen are represented in the simplified drug exposure diagram shown in Figure 3. We can clearly see that the fictitious patient is currently well below the desired therapeutic range, while the adjustment is expected to take the patient very close to the best therapeutic target. Detailed concentration-time profiles in plasma are given in Figure 4, where we can see what the concentration values have probably been thus far, as well as what the suggested dosing scheme is expected to deliver. The illustrative case is configured with the therapeutic trough range of the Gotta model, from 750 µg/L to 1500 µg/L, with a best therapeutic target of 1000 µg/L. The trough of 1067.72 µg/L predicted under the recommended regimen, shown in Figure 3, is the value the CDSS retains after evaluating every admissible regimen. Among the discrete set of allowed unit doses and dosing intervals, none attains the best target of 1000 µg/L exactly, so the system selects the regimen whose predicted trough lies closest to it. This range is the one declared in the Gotta model and differs from the 1100 µg/L to 2000 µg/L range configured for the authors’ own oncology deployment (see the Introduction section). The demonstrated recommendation therefore targets the model’s range, and its value below the deployment range reflects the different range in use rather than an underdosing.

**Figure 2.**
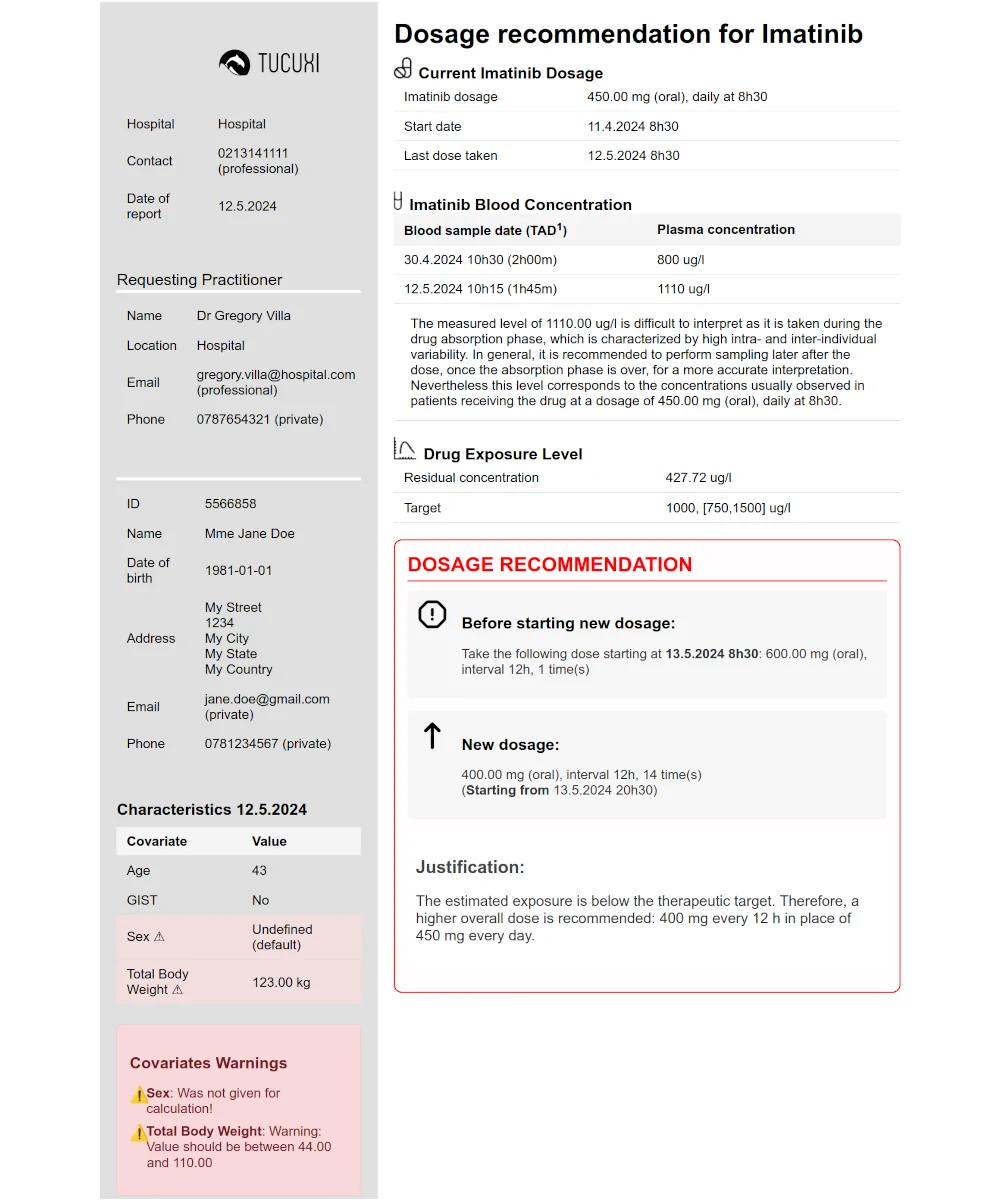
Textual section of the Tucuxi-CDSS report generated for a fictitious imatinib patient. The graphical elements are shown separately in Figures 3 and 4. The case is entirely illustrative and corresponds to no real patient.

**Figure 3.**
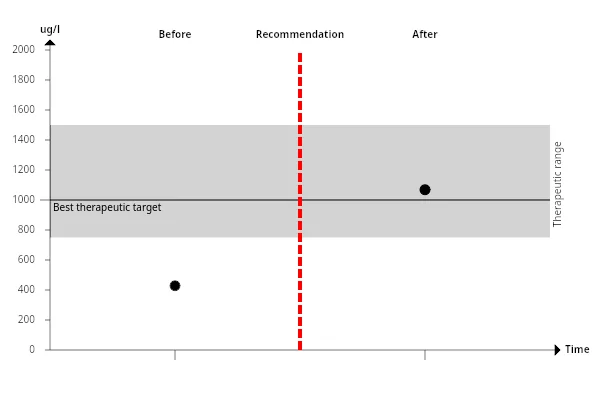
Example of a drug exposure diagram for the illustrative fictitious patient under imatinib, comparing the predicted exposure with the therapeutic target before and after the proposed dosage adjustment. For imatinib, the exposure metric is the trough concentration, reported on the vertical axis in µg/L. The shaded band is the therapeutic range declared in the drug model derived from Gotta et al [15,20], from 750 µg/L to 1500 µg/L, and the horizontal line is the best therapeutic target of 1000 µg/L. The left marker is the trough predicted under the current regimen of 450 mg every 24 hours, which lies below the therapeutic range; the right marker is the trough predicted under the recommended regimen of 400 mg every 12 hours, namely 1067.72 µg/L, close to the best target.

**Figure 4.**
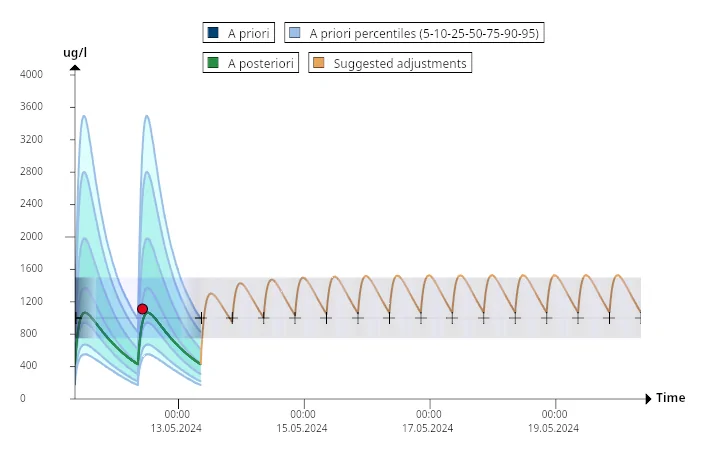
Example drug concentration-time profiles in plasma over time for the fictitious patient under imatinib treatment. It displays the therapeutic range (shaded gray areas, 750‐1500 µg/L, with 1000 µg/L as best), the predicted concentration-time profile (green line) and associated percentiles (blue lines and shaded area; 5th, 10th, 25th, 50th, 75th, 90th, and 95th) on the left, and the concentration-time profile (orange line) computed from the suggested adjustment on the right. The red dot marks the most recent observed concentration, drawn on May 12, 2024 (12.05.2024) during the absorption phase at an inappropriate time after the dose and flagged as such in the report of Figure 2. For legibility, only the last 2 dosing intervals preceding the report are plotted, so the earlier sample from April 30, 2024 lies before the plotted window and is not shown.

## Discussion

This work presented Tucuxi-CDSS, an open-source CDSS for MIPD whose distinguishing contribution is not the PK computation, which it delegates in its entirety to the Tucuxi-Core engine [23], but the validation layer interposed between the clinical record and that engine. Before any computation is undertaken, every covariate, dosage record, and concentration measurement is subjected to the configurable plausibility and consistency checks enumerated in Table 1, which render explicit and reproducible the scrutiny a TDM expert would otherwise apply by inspection; a decision layer then converts the resulting distribution of exposures into a single justified recommendation. Verification was conducted at the unit, system, and fuzzing levels. The *in silico* study reported in the companion paper [19] assessed the system on this basis: Tucuxi-CDSS matched a human TDM expert in error detection capability and brought significantly more patients to the therapeutic target, at the cost of 4 adjustments that displaced patients already within it.

### Principal Findings

Four findings emerged from the design and the assessment of the system. First, the “garbage in, garbage out” vulnerability of MIPD, long identified as the principal obstacle to its deployment outside expert centers, proves amenable to a systematic, rule-based treatment: The checks enumerated in Table 1 capture, at the software layer, a substantial share of the pre-analytical and data entry errors that a human expert would otherwise intercept by inspection. Second, this capability is not merely theoretical, and its shape is instructive: Against the expert, the system proved more sensitive but less specific, which is to say it intercepted more of the true errors and also raised more flags on data that were sound. The full comparison is summarized in the Methods section and reported in [19]. Third, and consequentially, the residual failure mode of such a system is not computational but evidential: 4 patients initially within the therapeutic window were displaced outside it following a CDSS recommendation, a class of intervention the expert did not produce. In one further case, the patient drifted out of target in both arms, the nature of the error having precluded any adaptation by either party. Those 4 cases are the price of active optimization, and the companion paper [19] weighs them in the terms that matter: Only 22% of the cohort lay within the target at baseline, so that the relevant comparison is not with a faultless intervention but with the therapeutic inertia that leaves most patients off target and undisturbed, and the resulting exposures, although outside the strict window, remained within a range generally tolerated for imatinib [18]. What relates to this paper is their mechanism. Each arose where the validation layer failed to intercept a corrupted input, and the reason is legible in the design set out earlier in this paper: The plausibility check compares a sample against the percentile envelope predicted for the typical patient, which is the criterion in routine clinical use, yet a single measurement seldom suffices to distinguish analytical noise from a genuine physiological shift. The system then optimizes confidently upon a datum it had no means of refusing. Its aggressiveness is only as safe as the data upon which it operates, which motivates rather than undermines the emphasis that this paper places upon the validation layer and which identifies where that layer must be strengthened: a module engineered specifically for error detection, operating upon richer criteria than percentile boundaries, as we record in the Future Directions section.

A fourth finding, of an engineering rather than a clinical nature, concerns configurability. By externalizing every drug-specific and institution-specific behavior into declarative XML, the system separates the question of what the software computes from the question of what a given clinical context considers acceptable. The therapeutic range, the covariate boundaries beyond which a model is deemed inapplicable, the percentile thresholds that distinguish an implausible sample from an unusual one, and the natural language in which the resulting recommendation is expressed are all deployment parameters rather than code. It is this separation that permitted a single executable to serve the rifampicin implementation study in Tanzania and the imatinib POC workflow in oncology without a line of C++ source code being altered between them.

### Comparison With Prior Work

A recent scoping review identified 28 software tools that may properly be called MIPD platforms, of which fewer than 20 remain available [25]. Their common denominator is a Bayesian estimation engine coupled to a library of popPK models, and their common assumption is that the person operating them is competent to judge the credibility of the data with which they are fed. Tucuxi-CDSS does not compete with these tools upon the ground of estimation quality; it delegates that task to Tucuxi-Core [23], whose numerical behavior has itself been independently verified [21]. What distinguishes the present system is the interposition of an explicit, configurable, rule-based validation layer between the clinical record and the estimation engine and of a decision layer between the estimation engine and the clinician. The former converts the implicit scrutiny of the expert into an explicit and reproducible artifact; the latter converts a distribution over exposure metrics into a single, justified, natural-language recommendation. Systematic reviews of MIPD implementation have converged upon precisely these 2 capabilities—user-facing tools whose outputs are intelligible without pharmacometric training and mechanisms for establishing the credibility of the underlying data and models—as the facilitators most likely to carry MIPD from the pharmacometric literature to the bedside [4,26].

The system is further distinguished by its open-source availability [24]. The limited availability of data for model validation and the absence of an agreed procedure for model endorsement are recurrently cited among the principal barriers to the adoption of MIPD [26]. A closed system can assert that its checks are sound; an open one can be inspected, independently reverified, and extended with new models or safeguards by the very parties who bear the clinical risk of using it. We regard this as a substantive property of the architecture rather than a licensing preference.

### Electronic Health Record Interoperability

Tucuxi-CDSS is delivered as a standalone command-line executable and equivalently as a linkable C++ library. It implements no health care interoperability standard: it neither consumes Health Level Seven (HL7) messages nor exposes a Fast Healthcare Interoperability Resources (FHIR) endpoint. Rather, it exposes a stable, well-typed XML input and output contract, which a thin institution-specific adapter is intended to bridge to whichever substrate the local environment provides. Such an adapter subscribes to the relevant clinical events, transforms them into a CDSS input document, invokes the executable, and publishes the returned recommendation together with its accompanying warnings back into the clinical workflow. The principal virtue of this arrangement is that the auditable surface of the system remains a single, versioned executable whose behavior is fully determined by its configuration files, while authentication, provenance recording, alert routing, and clinician-in-the-loop confirmation remain matters of institutional policy, implemented where institutional policy is properly implemented.

Tucuxi-CDSS is part of a wider ecosystem. Tucuxi-GUI, the graphical interface originally developed to allow pharmacologists to perform MIPD, exchanges data in 2 distinct formats and has been connected to institutional systems in production, namely a modular open laboratory information system (MOLIS) at the University Hospital of Lausanne and an IntelliSpace Critical Care and Anesthesia (ICCA) system at the Hospices Civils de Lyon. Interoperability with institutional information systems has therefore been demonstrated at the ecosystem level, though not yet for Tucuxi-CDSS itself.

A web-based deployment of Tucuxi-CDSS [27] is currently under development and approaches the integration question from a different direction. In this architecture, the computation is centralized upon a server, while a local client retains the identifying information of the patient. Only the quantities that the model actually requires—the covariate values, dosing history, and measured concentrations, stripped of every direct identifier—are transmitted over the network, and the report returned by the server is reassociated with the patient locally, therefore avoiding data privacy issues, should the server be compromised. Centralizing the computation concentrates the maintenance of drug models, covariate boundaries, and reporting templates in a single location, so that a correction or a model update propagates to every deployment without the redistribution of an executable; the data minimization discipline just described keeps the identifying information upon the machine where it was entered. This arrangement is intended to serve institutions that can neither install nor maintain a local binary, which is precisely the circumstance of the decentralized and resource-limited settings for which the system was conceived.

### Clinical Safety and Responsibility

A configurable CDSS intended for use in settings where a TDM expert may not be available raises safety and ethical questions that deserve to be addressed explicitly, rather than left to the inference of the reader.

Tucuxi-CDSS is a decision support tool, not a decision replacement tool. Every recommendation it produces is accompanied by the covariates and the samples upon which it was computed, by the warnings that the validation layer emitted, and by a natural-language justification of the proposed regimen. In any regulated health care context, that recommendation is to be reviewed and formally accepted by a licensed clinician who retains clinical responsibility for the dosing decision. The system is designed to render such review possible: Its report is legible, its warnings are stated in terms upon which a clinician without pharmacometric training can act, and its rationale is exposed rather than concealed behind a numeric output.

The treatment of covariates falling outside the validated boundaries of the population model merits particular attention, and the illustrative report in Figure 2 exhibits precisely this situation: The body weight of the fictitious patient exceeds the boundary declared in the imatinib drug file, the validation layer emits the corresponding warning in the covariate section of the report, and the system nonetheless proceeds to compute and present a dosage recommendation. This behavior reflects a considered choice between 2 failure modes. Refusing to compute leaves the clinician with no decision support whatever and, in practice, invites unstructured and undocumented dosing precisely in those patients whose physiology departs most from the population from which the model was estimated. Computing without warning invites undue confidence in an extrapolated estimate. The present design occupies the middle position: It computes, and it declares that it has extrapolated.

We acknowledge that the middle position carries a residual risk and that the risk is proportional to the visibility of the warning. A covariate warning displayed in one section of a report, although the recommendation appears in another, relies upon the clinician reading the document in full. This is a reasonable expectation of a clinician formally accepting a dosing recommendation, but it is not a guarantee, and the burden of the extrapolation falls upon a reader who may not be equipped to weigh it. Raising the salience of extrapolation warnings within the dosage recommendation block itself is, in our judgment, a worthwhile refinement of the reporting template, and we identify it as such in the Future Directions section. It would require no change to the computational core, only to the template that the system already treats as a deployment parameter.

Responsibility, in the deployment pattern we described here, distributes along the same lines as for established CDSS tools in oncology and in infectious disease. The developer answers for the correctness of the implemented algorithms and for the fidelity of their documented behavior; the deploying institution answers for the local configuration, which is to say, for the covariate boundaries, therapeutic targets, alert thresholds, and reporting templates that it chooses to install; and the prescribing clinician answers for the clinical decision taken upon the basis of the recommendation. The externalization of configuration into declarative files is not incidental to this distribution but constitutive of it: It renders the clinical policy of the institution an explicit, versionable, reviewable artifact rather than an assumption buried in source code.

Tucuxi-CDSS is a research tool. It is not certified for clinical use, and the deployments described in this paper are conducted within the framework of research projects rather than of routine care delivery. Its use outside such a framework would require an evaluation that lies beyond the scope of this work. That evaluation is not, however, closed to those who would undertake it. Because Tucuxi-CDSS is open source, a health institution may inspect its source, build and deploy it as in-house software, and subject it to whatever internal validation its own governance requires, thereby assuming full responsibility for its use.

### Limitations

This work is subject to several limitations.

First, the evidence supporting the clinical performance of the system is *in silico*. The evaluation reported in [19] used 60 virtual patients simulated from a population model and deliberately enriched with data-quality errors; it demonstrates that the system behaves as designed under controlled perturbation, not that it improves patient outcomes in practice. No prospective clinical evidence is yet available. The rifampicin implementation study currently underway in Tanzania is intended to generate the first such evidence, and its design accordingly emphasizes feasibility and safety before effectiveness.

Second, the safety of the system is contingent upon data integrity in a manner that its aggregate performance figures can obscure. The 4 detrimental interventions observed in [19] originated in errors the validation layer failed to detect (an extrapolation case, a misclassified steady state, a rounding of the sampling time, and a mislabeled sample). In one further case, a patient drifted out of the therapeutic range at steady state, with no adaptation possible in either arm. A validation layer that catches a majority of errors and then acts confidently upon the remainder is not unambiguously safer than one that catches none and acts timidly. The mitigation is the retention of the clinician as the terminal authority upon every recommendation, together with the visibility of the warnings upon which that authority is to be exercised.

Third, the quality of every recommendation is bounded by the quality of the underlying population model, over which the CDSS exercises no judgment. The system enforces the boundaries declared in the drug file; it cannot detect that a model is misspecified within them nor that the population from which it was estimated differs materially from the patient before it. This limitation is intrinsic to MIPD rather than particular to this implementation, and it argues for the discipline of model qualification advocated in the recent literature [4,14] rather than for any change to the software.

Fourth, the illustrative imatinib report in Figure 2 is constructed upon a fictitious patient. It walks the reader through the visible outputs of the system and demonstrates the classes of issue that it flags; it does not, and cannot, constitute clinical validation, which resides in [19].

Fifth, the verification campaign, although structured across the unit, system, and fuzzing levels, is empirical rather than exhaustive. Fuzzing establishes robustness against the perturbations it happens to generate; it offers no formal guarantee. No formal verification of the decision logic has been undertaken, but the proposed adjustments, at the heart of the system, are computed by Tucuxi-Core and have been thoroughly verified during the development of this library.

Sixth, no integration of the system into a production electronic health record has yet been carried out. The XML input and output contract is designed to be bridged to the local environment by an institution-specific adapter, and the aforementioned web-based deployment is under development, but neither have been evaluated in a clinical setting. The feasibility, latency, and clinical acceptability of such an integration remain to be demonstrated in practice.

Seventh, the alert fatigue properties of the system have not been measured. Its configurable thresholds are designed to permit an institution to tune the trade-off between sensitivity and alert volume; however, no empirical study has yet established where that threshold should sit for any given deployment, and an alert that is dismissed reflexively affords no protection whatever [11,12].

### Future Directions

Several extensions follow directly from the aforementioned limitations. The prospective evaluation of the system within the TuberXpert implementation study will provide the first evidence of its behavior upon real patients, in the setting for which it was originally conceived. The clinical, PK, and contextual data being collected in that study will further permit a retrospective characterization of the classes of pre-analytical issues that Tucuxi-CDSS actually encounters in routine practice, complementing the controlled *in silico* evidence of [19] with an observational counterpart drawn from the very population that the system is meant to serve.

On the validation side, the 4 detrimental interventions reported in [19] mark where the architecture is weakest. The present plausibility check flags a sample falling outside the percentile envelope predicted for the typical patient; this is the criterion in routine clinical use, and it is deliberately conservative. However, it cannot separate analytical noise from a true physiological change upon the evidence of a single measurement. A module engineered specifically for error detection, operating upon richer criteria than percentile boundaries, is, in our judgment, the principal avenue by which the safety of the system may be improved, and it is the direction we intend to pursue.

On the reporting side, 3 refinements suggest themselves, none of which requires a change to the underlying PK algorithms. Raising the salience of extrapolation warnings within the dosage recommendation block, as aforementioned, would reduce the reliance upon the clinician reading the report in full. Accompanying the predicted exposure metric of the recommended regimen, whichever quantity the drug model declares its target in, with its Bayesian prediction interval, derived from the a posteriori parameter distribution, would allow the clinician to appreciate the uncertainty attached to the estimate rather than reading a point value alone. In addition, replacing the current qualitative, percentile-based flagging of implausible samples with a quantitative report of the standardized residual between the observed and the predicted concentration, compared against a configurable threshold, would furnish a numerical, rather than a categorical, assessment of the local reliability of the model for the patient at hand, allowing the system to state explicitly that it appears to be under- or overestimating exposure in this particular individual.

On the decision side, the current output presents a single recommended regimen. Presenting, instead, a small set of admissible regimens, ranked by the adjustment quality score defined in the Methods section and annotated with their predicted exposure and compatibility with practical constraints such as the available galenic forms and an acceptable dosing frequency, would furnish the clinician with actionable alternatives when the top-ranked regimen conflicts with circumstances that the system cannot see.

Finally, the web-based deployment under development will require an evaluation of its own, both of the sufficiency of its data minimization scheme and its behavior under the network conditions of the settings that it targets. An adapter bridging the system to the interfaces of a production electronic health record would, in parallel, allow the aforementioned interoperability provisions to be demonstrated rather than merely asserted.

### Conclusions

This work presented the architecture, operational workflow, and configurability of Tucuxi-CDSS, an open-source CDSS designed to safely operationalize MIPD in routine clinical practice. By combining extensive data validation, automated plausibility checks, Bayesian forecasting through the Tucuxi-Core engine, and configurable decision rules, the system provides a structured framework to better detect pre-analytical errors and enhances the reliability of dosing recommendations. Its modular design allows institutions to adapt drug models, therapeutic targets, language settings, and reporting templates to local needs, ensuring broad applicability across diverse health care environments.

A key strength of Tucuxi-CDSS is that it brings structured, reproducible TDM oversight to settings where trained pharmacologists and TDM experts are scarce—a requirement highlighted both in resource-limited contexts such as the TuberXpert TB initiative and in decentralized oncology workflows using POC biosensing. Its open-source nature [24] further enables community-driven development, external validation, and the transparent integration of new popPK models or safeguards. Integrable either as a standalone backend or as a library within existing clinical software infrastructures and, in a web-based deployment currently under development, as a centralized service preserving the locality of identifying data, Tucuxi-CDSS provides a foundation for the safe, scalable, and reproducible implementation of precision dosing at the bedside.

Its performance evaluation against a TDM expert, summarized in this manuscript and reported in full in the companion paper [19], paves the way for the prospective studies that must precede the adoption of such a tool in clinical practice.

## Abbreviations

CDSS: clinical decision support system
FHIR: Fast Healthcare Interoperability Resources
GIST: gastrointestinal stromal tumor
HL7: Health Level Seven
ICCA: IntelliSpace Critical Care and Anesthesia
MIPD: model-informed precision dosing
MOLIS: modular open laboratory information system
PD: pharmacodynamic
PK: pharmacokinetic
POC: point-of-care
popPK: population pharmacokinetics
TB: tuberculosis
TDM: therapeutic drug monitoring
